# Effects of photobiomodulation, alone or combined with dexamethasone, on cellular metabolic activity and inflammatory markers in LPS/IFN-γ-stimulated J774 macrophages

**DOI:** 10.1007/s10103-026-04978-2

**Published:** 2026-08-03

**Authors:** Rosani Tereza de Siqueira e Silva, Alessandra Lima da Silva Martins, Tainá Caroline dos Santos Malavazzi, Mohammadhossein Shaker, Natalia Cristina Cova de Souza, Aline Silva Souza, José Antonio Silva Junior, Sandra Kalil Bussadori, Anna Carolina Ratto Tempestini Horliana, Kristianne Porta Santos Fernandes, Raquel Agnelli Mesquita-Ferrari, Cinthya Cosme Gutierrez Duran

**Affiliations:** 1 Postgraduate Program in Biophotonics-Medicine, Universidade Nove de Julho, São Paulo, Brazil; 2 Department of Oral Pathology, School of Dentistry, Universidade de São Paulo, São Paulo, Brazil

**Keywords:** Photobiomodulation therapy, Macrophages, activated, Dexamethasone, Inflammation, Cytokines

## Abstract

The aim of the present study was to investigate the effects of photobiomodulation (PBM), dexamethasone (DEXA), and their combination on cellular metabolic activity and inflammatory cytokines in LPS/IFN-γ-stimulated J774 macrophages.Cells were stimulated with lipopolysaccharide (LPS) and interferon-gamma (IFN-γ) and treated with PBM (780 nm) and/or DEXA (2 or 4 μM). Cellmorphology, metabolic activity assessed by the 3-[4,5-dimethylthiazol-2-yl]-2,5-diphenyltetrazolium bromide (MTT) assay, and total protein, tumornecrosis factor-alpha (TNF-α), and interleukin-6 (IL-6) levels measured by enzyme-linked immunosorbent assay (ELISA) were analyzed at 24 and 48hours. PBM combined with DEXA at 2 μM increased MTT metabolic activity at 24 hours and was associated with preserved morphology and maintainedhigher total protein levels, with consistent superiority over DEXA alone. PBM + DEXA 2 μM produced the strongest reduction in TNF-α and IL-6 at 24hours, and IL-6 levels remained lower at 48 hours compared to the M1 + PBM group. The 4 μM dose demonstrated lower efficacy and signs of functionalimpairment, which were partially mitigated by PBM. These findings indicate that PBM may enhance the anti-inflammatory effects of low-dose DEXA while supporting a favorable cellular metabolic response, suggesting its potential as an adjunct strategy to optimize the glucocorticoid-based modulation ofinflammatory responses.

## Highlights

Photobiomodulation combined with dexamethasone was investigated in LPS/IFN-γ-stimulated J774 macrophages.

The combination of photobiomodulation and a lower dexamethasone concentration was associated with the modulation of pro-inflammatory cytokines.

Photobiomodulation combined with a lower dexamethasone concentration was associated with a favorable cellular metabolic response in the experimental model.

The findings suggest photobiomodulation as an adjunct approach to corticosteroid-based anti-inflammatory strategies.

## Introduction

Inflammation is an essential immune response to infection and tissue injury. However, persistent or dysregulated activation may lead to a chronic inflammatory state, as occurs in several diseases [1, 2]. Macrophages are key effector cells of the innate immune system that contribute to host defense and tissue remodeling through phagocytosis and the secretion of inflammatory mediators [3–5].

Macrophages display substantial functional plasticity and can polarize into distinct phenotypes. The M1 phenotype is characterized by the production of tumor necrosis factor-alpha (TNF-α), interleukin (IL)-1β, IL-6, and inducible nitric oxide synthase (iNOS), which are mediators involved in defense against microbial attack, but can also promote tissue damage when produced in excess [1–3]. In contrast, M2 macrophages secrete IL-10 and transforming growth factor-beta (TGF-β), facilitating the resolution of inflammation and tissue repair [1–3]. Owing to this dichotomy, regulating the balance between the M1 and M2 phenotypes has become an important therapeutic target in chronic inflammatory disorders [1–3].

Photobiomodulation (PBM) has emerged as a noninvasive therapeutic modality capable of modulating inflammatory pathways and promoting tissue regeneration. Its mechanisms include photon absorption by mitochondrial chromophores, particularly cytochrome c oxidase, resulting in increased adenosine triphosphate synthesis and the modulation of signaling pathways, such as nuclear factor kappa-light-chain-enhancer of activated B cells (NF-κB) and mitogen-activated protein kinase (MAPK) [6–8]. Recent evidence indicates that PBM modulates redox balance, enhances mitochondrial activity, and suppresses pro-inflammatory cytokine signaling, thus playing a role in regulating the immune response [9–12].

In macrophages, the biological response to PBM appears to depend on the activation state of the cell, the inflammatory microenvironment, and the irradiation parameters applied. In activated macrophages, PBM has been associated with modulation of mitochondrial metabolism, redox signaling, and inflammatory mediator production, which may contribute to the regulation of excessive inflammatory responses [6–9, 11, 12]. These effects are particularly relevant in experimental models involving stimulated macrophages, in which the balance between cellular metabolic activity and cytokine production is essential for evaluating potential anti-inflammatory strategies.

Experimental findings have demonstrated the anti-inflammatory activity of PBM in macrophage models. Fernandes et al. showed that irradiation with 660-nm and 780-nm lasers reduced TNF-α, IL-6, and iNOS levels in activated J774 cells [11]. In addition, previous studies have suggested that PBM may influence macrophage functional phenotype and inflammatory mediator expression, supporting its potential role as a physical strategy for modulating macrophage-driven inflammatory responses [9, 16, 19].

Dexamethasone (DEXA) is a potent synthetic glucocorticoid that suppresses inflammatory signaling by inhibiting NF-κB activation and decreasing the transcription of pro-inflammatory cytokines, such as TNF-α and IL-6 [13, 14]. However, prolonged or high-dose exposure may induce apoptosis and increase oxidative stress [15], potentially limiting its long-term clinical utility. Thus, the combination of PBM and DEXA may constitute a complementary therapeutic approach, as PBM could attenuate glucocorticoid-induced cytotoxicity while enhancing anti-inflammatory efficacy [16–20].

The aim of the present study was to investigate the in vitro effects of PBM, DEXA, and their combination on cellular metabolic activity and inflammatory cytokine expression in LPS/IFN-γ-stimulated J774 macrophages.

## Materials and methods

**Fig. 1:**
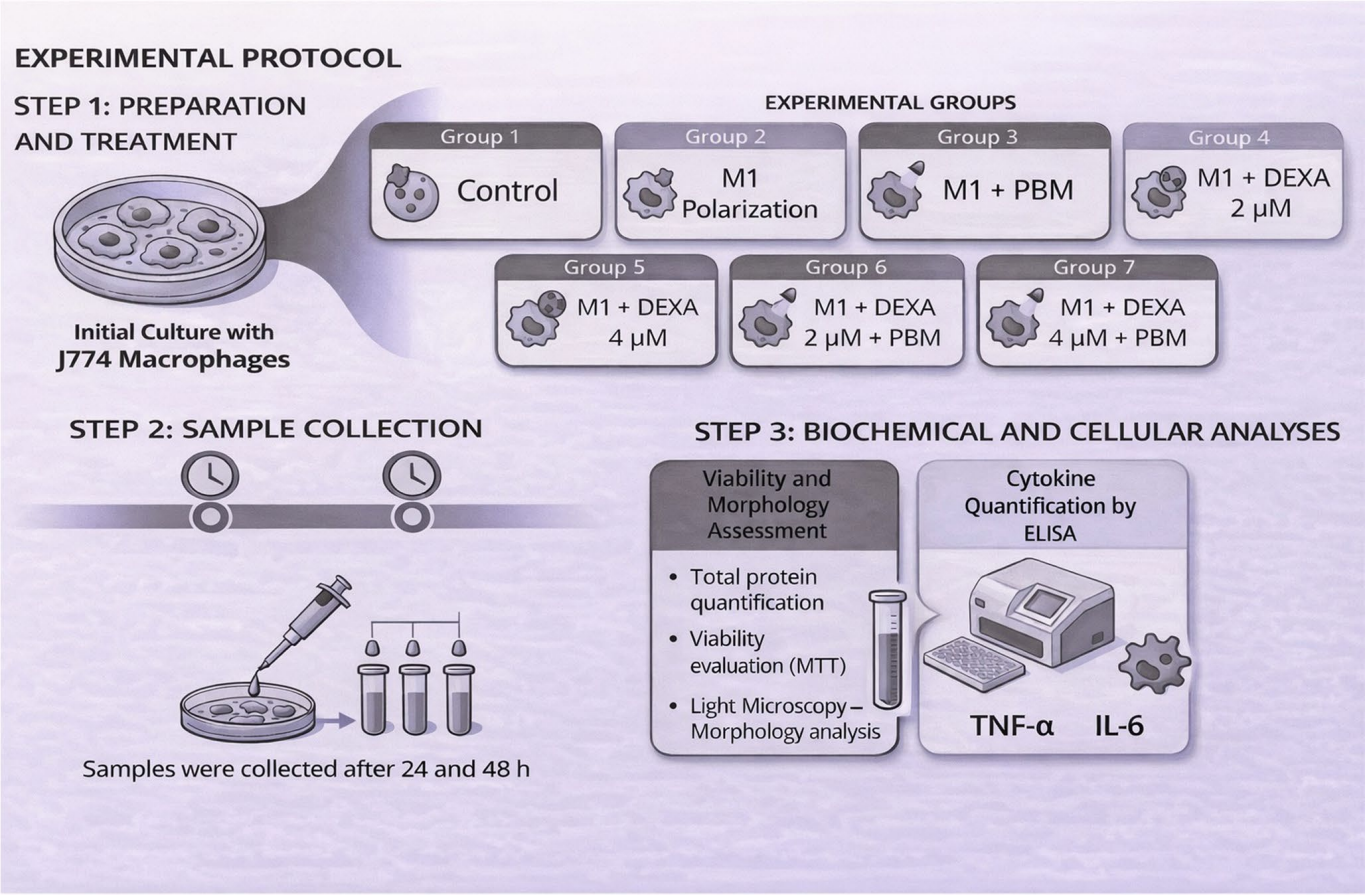
Experimental Protocol. Schematic representation of study design, including LPS + IFN-γ stimulation, photobiomodulation (PBM) and dexamethasone (DEXA) treatments, incubation periods (24 h and 48 h), and subsequent analyses: morphological assessment, cellular metabolic activity assessed by the MTT assay, total protein quantification, and cytokine measurement, including TNF-α and IL-6, by enzyme-linked immunosorbent assay (ELISA). The figure was created using BioRender.com, with AI-assisted design features, based on a conceptual layout developed by the authors, who retain full responsibility for the scientific content, accuracy, and integrity of the figure

The experimental design and treatment protocol are illustrated in Fig. 1.

### Macrophage culture and LPS/IFN-γ stimulation

J774 murine macrophage cells were maintained in Dulbecco’s Modified Eagle’s Medium (DMEM) supplemented with 10% fetal bovine serum (FBS), penicillin (100 U/mL), and streptomycin (100 µg/mL). Cultures were incubated at 37 °C in a humidified atmosphere containing 5% CO₂. For the experiments, cells were seeded in 24-well plates at a density of 1 × 10⁵ cells per well.

Macrophage stimulation was induced by incubating the cells with lipopolysaccharide (LPS, 1 µg/mL) and interferon-gamma (IFN-γ, 0.2 µg/mL) for 2 h, based on protocols previously used in murine macrophage models to induce a pro-inflammatory activation state [11, 19, 20]. After the stimulation period, cells were washed four times with 1X phosphate-buffered saline (PBS) to remove the stimulating agents before the application of the experimental treatments.

### Experimental groups

The experimental groups were organized as follows. The term M1 was used operationally to identify the group stimulated with LPS plus IFN-γ:

- Control (non-activated, no treatment)
- M1 (activated, no treatment)
- M1 + PBM
- M1 + DEXA at 2 µM
- M1 + DEXA at 4 µM
- M1 + PBM + DEXA at 2 µM
- M1 + PBM + DEXA at 4 µM

### Photobiomodulation (PBM)

Photobiomodulation was applied with infrared laser (780 nm, 70 mW, 26.25 J/cm², total energy of 1.05 J, continuous mode) using the Twin Laser device (MM Optics, São Carlos, SP, Brazil). Irradiation was delivered as a single spot directed onto the cell pellet with an exposure time of 15 s. The complete dosimetric parameters used in this study are presented in Table 1.

**Table 1:** Dosimetric parameters used for PBM treatment

Parameter	Laser
Wavelength (nm)	780
Spectral bandwidth (nm)	10
Emission mode	Continuous
Output Power(mW)	70
Polarization	Random
Aperture diameter (cm)	0.23
Irradiance at the aperture (mW/cm²)	1750
Beam area (cm²)	0.04
Exposure time (s)	15
Number of irradiation points	1
Radiant exposure (J/cm²)	26.25
Irradiated area (cm²)	0.04
Application mode	Contact
Number of sessions and frequency	1
Total energy delivered (J)	1.05

The PBM parameters used in the present study were selected based on previous experimental studies involving J774 macrophages and near-infrared irradiation, in which similar PBM conditions were associated with modulation of inflammatory mediators without evident cytotoxic effects [11, 19]. As shown in Table 1, the radiant exposure was 26.25 J/cm², corresponding to a total delivered energy of 1.05 J over an irradiated area of 0.04 cm².

### Dexamethasone treatment

After the 2-hour stimulation period with LPS plus IFN-γ, macrophage cultures were washed four times with 1X phosphate-buffered saline (PBS) to remove the stimulating agents. The DEXA used in this study consisted of dexamethasone sodium phosphate and excipients, including sodium metabisulfite, dibasic sodium phosphate, monobasic sodium phosphate, and water, from an injectable formulation provided by Aché Laboratórios Farmacêuticos S.A. (São Paulo, SP, Brazil).

A stock solution was prepared in DMEM at a concentration of 100 µM. Working solutions of 2 µM and 4 µM were obtained by direct dilution of the stock solution in the culture medium. For treatment, 40 µL of the 2 µM DEXA solution or 80 µL of the 4 µM DEXA solution were added to the corresponding experimental groups.

The experimental sequence was as follows: at t = 0 h, macrophages were stimulated with LPS plus IFN-γ; at t = 2 h, cells were washed four times with 1X PBS; immediately after washing, DEXA was added to the corresponding groups and PBM was applied to the irradiated groups. Cells then remained continuously exposed to DEXA during the subsequent incubation period and were analyzed after 24–48 h.

Because the DEXA used in this study was obtained from a commercially available injectable pharmaceutical formulation, the excipients present in the formulation, including sodium metabisulfite, were also present in the DEXA-treated groups. Although this approach reflects the use of the complete pharmaceutical formulation, an excipient-only control was not included. Therefore, a possible contribution of the formulation excipients to the observed responses cannot be completely excluded and is acknowledged in the Discussion as a study limitation.

### Morphological analysis

Cell morphology was assessed two, 24, and 48 h after treatment. All experimental groups were imaged using a ZOE™ Fluorescent Cell Imager (Bio-Rad Laboratories, Hercules, CA, USA) equipped with a fixed 20× magnification for bright-field image acquisition. Cells were examined without fixation and maintained in the culture medium during imaging. The morphological assessment was performed qualitatively based on classic criteria of cell culture analysis, such as adhesion, cell spreading, confluence, cell shape, and monolayer organization.

### Cellular metabolic activity assay

Cellular metabolic activity was indirectly assessed using the 3-[4,5-dimethylthiazol-2-yl]-2,5-diphenyltetrazolium bromide (MTT) assay (Sigma-Aldrich, St. Louis, MO, USA). The MTT assay estimates the metabolic activity of viable cells through the reduction of tetrazolium salts by multiple cellular enzymatic systems, including but not limited to mitochondrial enzymes [21, 22]. Cells (1 × 10⁵ per well) from all experimental groups were cultured in flat-bottom 96-well plates (TPP, Trasadingen, Switzerland) and analyzed after 24 and 48 h of incubation. Each condition was analyzed in quadruplicate and all experiments were performed independently three times.

After the experimental periods, the supernatant of the culture was removed and the cells were washed with 1X PBS. Subsequently, 50 µL of MTT solution (0.5 mg/mL in phenol red–free DMEM) were added to each well and the plates were incubated for three hours at 37 °C in a humidified atmosphere containing 5% CO₂. After incubation, the supernatant was discarded and the formazan crystals were solubilized with 100 µL of isopropanol (Synth, Diadema, SP, Brazil). Absorbance was measured at 540 nm in a microplate reader (Anthos 2020^®^, Anthos Labtec Instruments, Wals, Austria). MTT metabolic activity was expressed as a percentage relative to the control group.

### Total protein quantification

Total protein concentration in the supernatants was determined after 24 and 48 h of incubation. Samples were collected, centrifuged at 1,000 g for 10 min, and analyzed by spectrophotometry using a NanoDrop 2000 device (Thermo Scientific, Waltham, MA, USA), with readings at 280 nm. Protein values (mg/mL) were used to normalize the cytokine concentrations obtained in the enzyme-linked immunosorbent assays (ELISA). All experimental groups were maintained with the same fetal bovine serum (FBS) concentration and medium volume. Because total protein was measured in culture supernatants, this parameter was not interpreted as a direct marker of intracellular metabolic activity or secretory function.

### Cytokine quantification by ELISA

Supernatants were collected after 24 and 48 h, centrifuged at 1,000 g for 10 min, and stored at − 80 °C until analysis. TNF-α and IL-6 concentrations were quantified using murine-specific ELISA kits (Mouse TNF-α ELISA MAX Deluxe Set and Mouse IL-6 ELISA MAX Deluxe Set, BioLegend^®^, San Diego, CA, USA), following the manufacturer’s instructions. Plate readings were performed in a microplate reader (Anthos 2020^®^, Anthos Labtec Instruments, Wals, Austria). Cytokine levels were normalized to the total protein concentration of each sample, as described in the previous section.

### Statistical analysis

Data were expressed as mean ± standard error of the mean (SEM). Normality was determined using the Kolmogorov–Smirnov test. One-way analysis of variance (ANOVA) followed by Tukey’s post hoc test was used for multiple group comparisons. Statistical significance was set at *p* < 0.05. All analyses were performed using GraphPad Prism version 8.01 (GraphPad Software, San Diego, CA, USA).

## Results

### Morphology

**Fig. 2:**
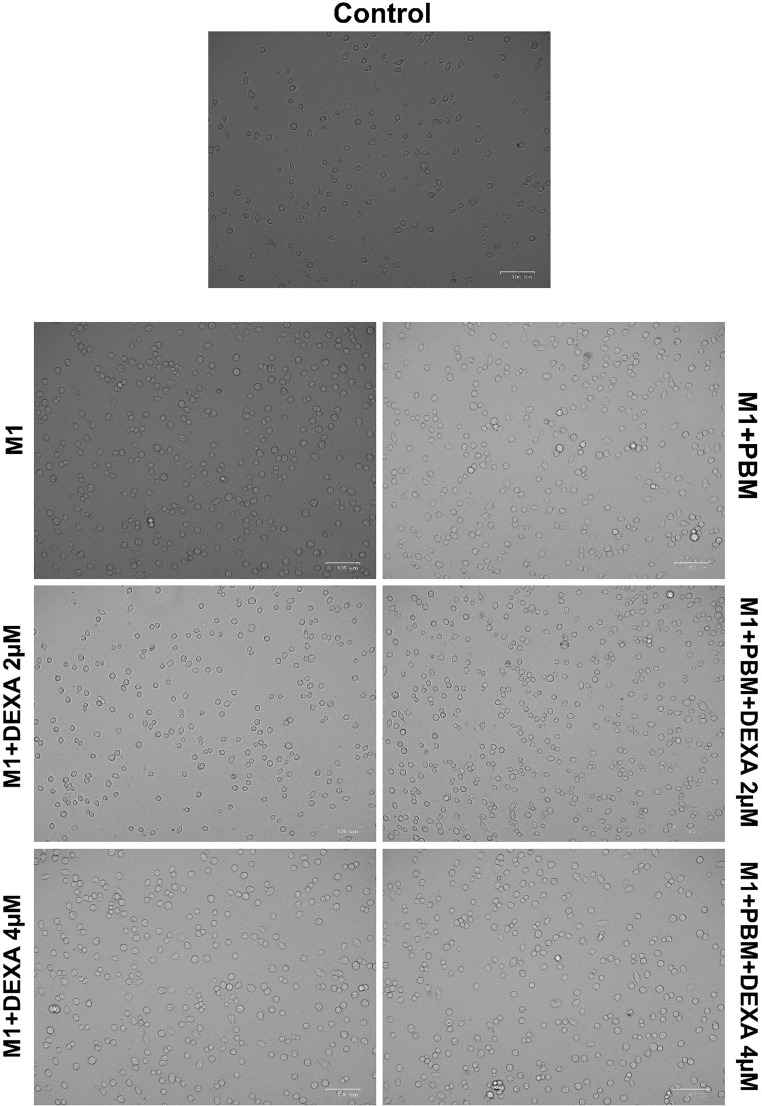
Representative images of cell morphology in experimental groups 48 h after treatment. J774 cells observed under bright-field microscopy using ZOE™ Fluorescent Cell Imager (Bio-Rad) with 20× magnification. Groups shown: Control, M1, M1 + PBM, M1 + DEXA 2 µM, M1 + DEXA 4 µM, M1 + PBM + DEXA 2 µM, and M1 + PBM + DEXA 4 µM

Representative images of the experimental groups after 48 h of incubation are shown in Fig. 2. The control group exhibited a homogeneous cell distribution and preserved morphology. Cells in the M1 group maintained a morphological pattern similar to that of the control. In the groups treated with DEXA, morphological changes were observed in a concentration-dependent manner. At 2 µM, slight cell rounding and the onset of monolayer irregularity were found. At 4 µM, a reduction in cell density (confluence), more pronounced rounding, and disruption of the monolayer were noted.

The groups treated with PBM exhibited morphology similar to that of the M1 group, with no relevant structural changes. In the groups treated with the combination PBM + DEXA, better preservation of confluence and greater monolayer uniformity were observed when compared to the respective groups treated with DEXA alone at both 2 µM and 4 µM. This difference was particularly evident at the 4 µM concentration, as PBM attenuated the loss of adhesion and reduced the fragmentation of the monolayer seen in the group treated with DEXA alone.

### Cellular metabolic activity assessed by MTT assay

**Fig. 3:**
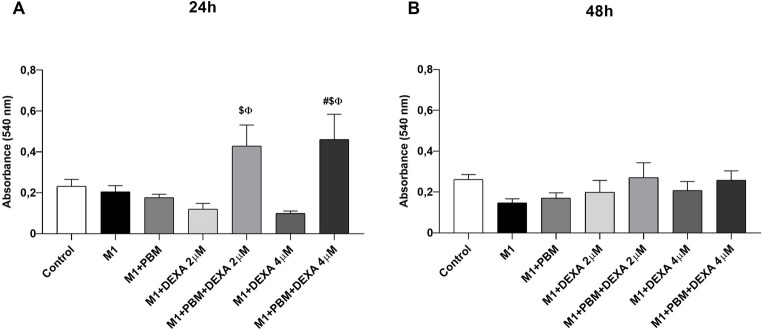
Cellular metabolic activity assessed by MTT assay in experimental groups after 24 h (**A**) and 48 h (**B**) of incubation. Data expressed as mean ± SEM. # *p* < 0.05 vs. PBM; $ *p* < 0.05 vs. DEXA 2 µM; Φ *p* < 0.05 vs. DEXA 4 µM (one-way ANOVA followed by Tukey’s test)

MTT metabolic activity results at 24 and 48 h are presented in Fig. 3. At 24 h (Fig. 3A), the M1 + PBM + DEXA 2 µM group exhibited greater MTT metabolic activity than the groups treated only with DEXA 2 µM (*p* = 0.015) and DEXA 4 µM (*p* = 0.015) without irradiation. The M1 + PBM + DEXA 4 µM group exhibited greater MTT metabolic activity relative to the M1 + PBM (*p* = 0.028), M1 + DEXA 2 µM (*p* = 0.006), and M1 + DEXA 4 µM (*p* = 0.007) groups. No significant differences were found between the PBM + DEXA 2 µM and PBM + DEXA 4 µM groups. Overall, the irradiated groups combined with DEXA exhibited greater MTT metabolic activity compared to the respective non-irradiated DEXA groups. At 48 h (Fig. 3B), no significant differences among the groups were found.

### Total protein quantification

**Fig. 4:**
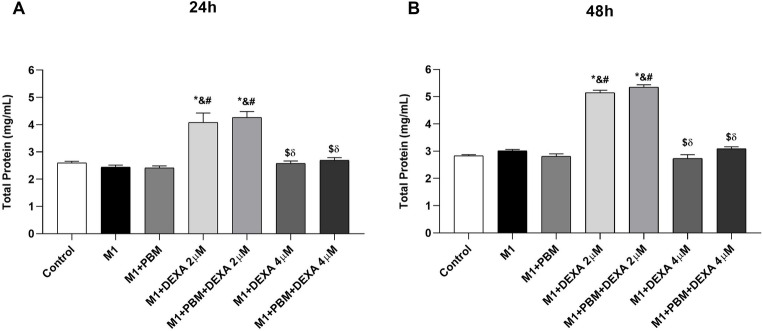
Quantification of total protein in supernatants of experimental groups after 24 h (**A**) and 48 h (**B**) of incubation. Data expressed as mg/mL (mean ± SEM). *p < 0.05 vs. control; & *p* < 0.05 vs. M1; # *p* < 0.05 vs. M1 + PBM; $ *p* < 0.05 vs. M1 + DEXA 2 µM; δ *p* < 0.05 vs. DEXA 2 µM + PBM (one-way ANOVA followed by Tukey’s post hoc test)

Total protein levels after 24 and 48 h of incubation are presented in Fig. 4.

At 24 h (Fig. 4A), the M1 + DEXA 2 µM groups with and without PBM had higher total protein levels compared to the control (*p* < 0.0001, both), M1 (*p* < 0.0001, both), and M1 + PBM groups (*p* < 0.0001, both). Moreover, the levels in the DEXA 2 µM groups with and without PBM were higher than those of the groups treated with DEXA 4 µM with and without PBM (*p* < 0.0001). This pattern was maintained at 48 h (Fig. 4B): groups treated with DEXA 2 µM with or without PBM had significantly higher total protein levels compared to all other groups (*p* < 0.0001).

### TNF-α levels

**Fig. 5:**
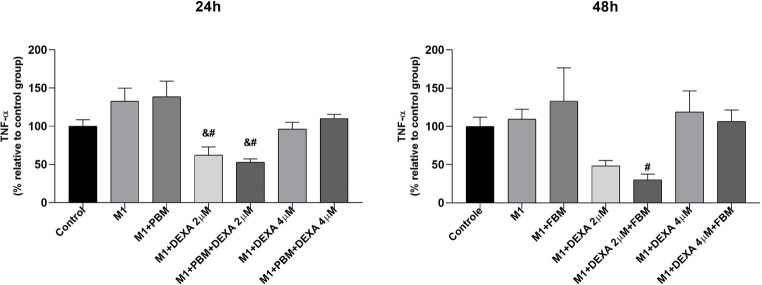
TNF-α levels (% relative to control group) after 24 h and 48 h of incubation. Data expressed as mean ± SEM. & *p* < 0.05 vs. M1; # *p* < 0.05 vs. M1 + PBM (one-way ANOVA followed by Tukey’s post hoc test)

TNF-α levels after 24 and 48 h of incubation are presented in Fig. 5.

At 24 h, the M1 + DEXA 2 µM group had lower TNF-α levels compared to the M1 and M1 + PBM groups (*p* = 0.0054 and *p* = 0.0022, respectively). These differences were more pronounced in the M1 + DEXA 2 µM + PBM group (*p* = 0.0013 and *p* = 0.0005, respectively), indicating an additional effect of irradiation when combined with the lower dexamethasone concentration.

At 48 h, the M1 + DEXA 2 µM + PBM group maintained significantly lower TNF-α levels compared to the M1 + PBM group (*p* = 0.045). No statistically significant differences were found among the other groups at this point.

### IL-6 levels

**Fig. 6:**
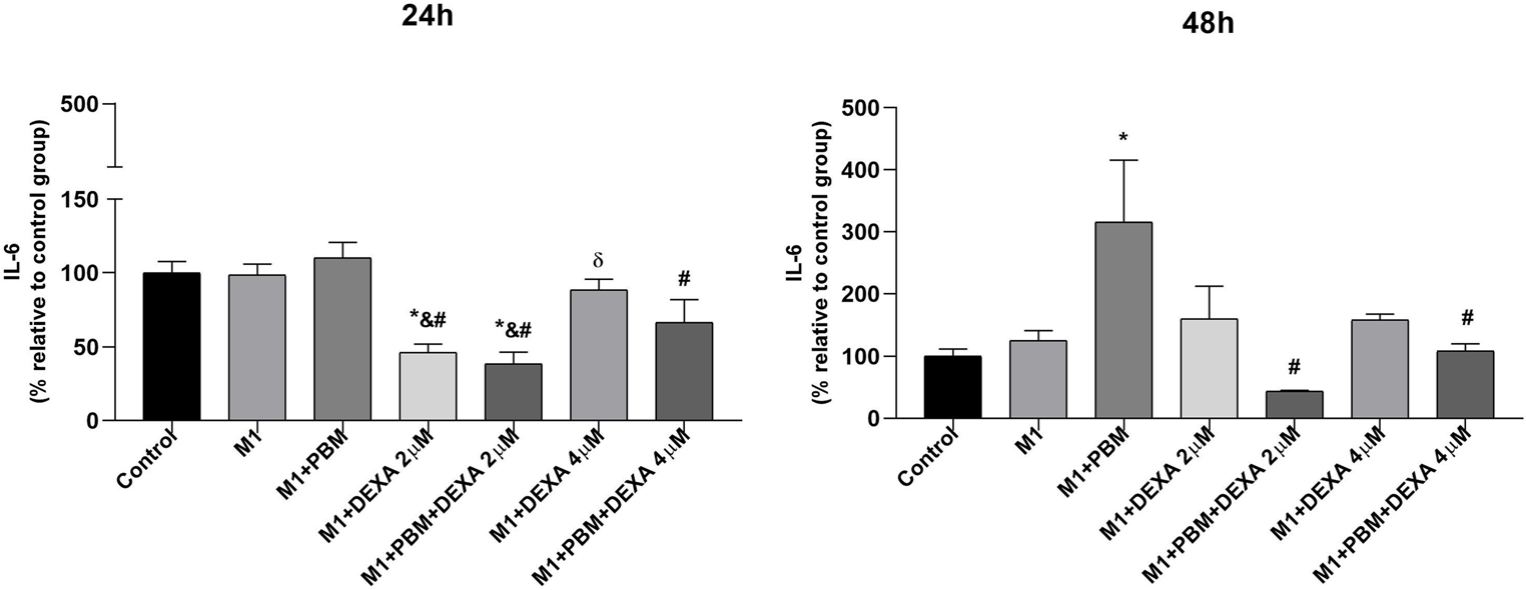
IL-6 levels (pg/mg total protein) as percentage of control group after 24 h and 48 h. Data expressed as mean ± SEM. *p < 0.05 vs. control; & *p* < 0.05 vs. M1; # *p* < 0.05 vs. M1 + PBM; $ *p* < 0.05 vs. M1 + DEXA 2 µM; δ *p* < 0.05 vs. DEXA 2 µM + PBM (one-way ANOVA followed by Tukey’s post hoc test)

IL-6 levels after 24 and 48 h of incubation are presented in Fig. 6.

At 24 h, the M1 + DEXA 2 µM and M1 + PBM + DEXA 2 µM groups had lower IL-6 levels compared to the control (*p* = 0.0083 and *p* = 0.0021, respectively), M1 (*p* = 0.011 and *p* = 0.0027, respectively), and M1 + PBM groups (*p* = 0.0014 and *p* = 0.0003, respectively). An increase was noted in the groups that received a higher concentration of DEXA (4 µM). Specifically, this effect was observed without PBM when compared to M1 + PBM + DEXA 2 µM (*p* = 0.016) and with PBM when compared to M1 + PBM (*p* = 0.044). No differences were found when comparing the M1 + DEXA groups at either concentration to their respective irradiated groups.

At 48 h, the M1 + PBM group exhibited higher IL-6 levels compared to the control group (*p* = 0.030) and the M1 + PBM + DEXA 2 µM group had significantly lower IL-6 levels than the M1 + PBM group (*p* = 0.0039). At the 4 µM concentration, PBM reduced IL-6 levels compared to the M1 + PBM group (*p* = 0.04).

## Discussion

Activation of macrophages toward a pro-inflammatory phenotype is recognized as a central component of the amplification and persistence of inflammatory responses and is classically associated with the expression of mediators such as TNF-α, IL-6, and iNOS following stimulation with LPS and interferon-gamma (IFN-γ) [1–4]. The combined use of LPS and IFN-γ is a commonly used in vitro stimulation protocol in murine macrophages, including J774 cells, and has been employed in previous studies investigating inflammatory mediator expression and the effects of photobiomodulation on macrophage responses [11, 19, 20].

In the present study, macrophage stimulation was performed using this dual-stimulation protocol. However, LPS + IFN-γ stimulation did not result in statistically significant increases in TNF-α and IL-6 levels in the M1 group compared with the control group at the evaluated time points. This finding indicates that the induction protocol did not produce a robust cytokine increase under the experimental conditions used and should therefore be interpreted as a limitation of the study.

Despite this limitation, the stimulated macrophages maintained functional responsiveness to the interventions, as low-dose dexamethasone, either alone or combined with photobiomodulation, modulated TNF-α and IL-6 levels in comparison with stimulated groups. Thus, the findings were interpreted more cautiously as results obtained in LPS/IFN-γ-stimulated J774 macrophages that remained responsive to anti-inflammatory modulation.

Within the context of anti-inflammatory therapies, dexamethasone remains one of the most widely used and effective pharmacological agents for suppressing pro-inflammatory mediators, primarily through the inhibition of NF-κB activation and the reduced transcription of TNF-α and IL-6 [13, 14]. In the present study, DEXA at the concentration of 2 µM significantly reduced TNF-α levels at 24 h (Fig. 5), indicating that the lower dose was able to modulate TNF-α levels in LPS/IFN-γ-stimulated macrophages. In contrast, the higher concentration (4 µM) did not promote additional reductions and exhibited less consistent effects, which is in agreement with evidence indicating that high glucocorticoid doses may increase oxidative stress and compromise cellular responses, thereby limiting anti-inflammatory efficacy [15].

Cellular metabolic activity was assessed using the MTT assay, which reflects the reduction of tetrazolium salts by metabolically active cells and should not be interpreted exclusively as a direct measure of cell number or viability. Therefore, changes in MTT absorbance should be interpreted as changes in cellular metabolic activity rather than as evidence of increased viability alone [21–23]. The combination of PBM and DEXA at 2 µM produced the most consistent increase in MTT metabolic activity at 24 h compared to the groups treated with DEXA alone (Fig. 3A). These findings are consistent with previous reports showing that PBM may support cellular metabolic responses [6–8, 16, 17] and modulate the expression of inflammatory mediators in activated macrophages [11, 19, 20]. At 48 h, no significant differences were found among groups (Fig. 3B), indicating that the differences observed at 24 h were not maintained at this later time point.

Although total protein quantification in the culture supernatant is not a direct measure of intracellular metabolic activity or secretory function, it provided complementary information on the overall extracellular protein profile under the different experimental conditions. In the present study, the groups treated with 2 µM DEXA, with or without PBM, exhibited higher total protein levels at both 24 and 48 h (Fig. 4), whereas lower values were observed in the groups treated with 4 µM DEXA. When interpreted together with the MTT and morphological findings, this pattern suggests a more favorable cellular response at the lower dexamethasone concentration. However, these data should be interpreted cautiously, since supernatant protein content may reflect multiple processes, including altered secretion patterns, protein release, or changes in cell integrity.

The modulation of TNF-α revealed a pronounced effect of the 2 µM DEXA dose and a relevant interaction with PBM. At 24 h, DEXA significantly reduced TNF-α only at the lower concentration, while PBM further enhanced this effect. At 48 h, TNF-α levels remained lower in the PBM + DEXA 2 µM group compared with the M1 + PBM group.

These findings are consistent with evidence [9, 10, 12], which reported that photobiomodulation may exert anti-inflammatory effects across experimental models using red and near-infrared wavelengths, with biological effects commonly observed between six and 48 h after irradiation. The present data indicate that PBM combined with low-dose DEXA constitutes a balanced strategy capable of reducing TNF-α while supporting a favorable cellular response. In this context, de Brito Sousa et al. demonstrated that red and infrared PBM differentially modulated inflammatory and anti-inflammatory mediators in M1 and M2 macrophages, supporting the role of PBM in macrophage functional regulation [19].

The modulation of IL-6 followed a concentration- and time-dependent pattern. At 24 h, both the M1 + DEXA 2 µM and M1 + PBM + DEXA 2 µM groups exhibited significantly lower IL-6 levels, whereas at 48 h, IL-6 levels remained lower in the PBM + DEXA 2 µM group compared with the M1 + PBM group. This behavior contrasts that of TNF-α and reflects the pleiotropic, context-dependent role of IL-6 in macrophage biology, which includes not only pro-inflammatory signaling but also regulatory functions related to cellular adaptation and resolution processes [3]. In addition, previous studies have shown that PBM may modulate inflammatory and anti-inflammatory mediators in macrophages, supporting its role in macrophage functional regulation [9, 19]. Accordingly, the selective modulation of IL-6 observed under the combined PBM + low-dose DEXA condition may indicate a favorable inflammatory profile associated with the cellular metabolic response observed in this group.

Taken together, these findings suggest that PBM combined with low-dose dexamethasone (2 µM) provides a more favorable cellular and inflammatory profile in LPS/IFN-γ-stimulated J774 macrophages than dexamethasone alone. This supports its potential relevance as an adjunct strategy for optimizing glucocorticoid-based modulation of inflammatory responses.

### Limitations

Some methodological aspects should be considered when interpreting the findings. This study was conducted in vitro using a murine macrophage cell line, which allows experimental control but does not fully reproduce the complexity of primary human macrophages. In addition, although LPS/IFN-γ stimulation was used to induce a pro-inflammatory condition, TNF-α and IL-6 levels in the stimulated M1 group were not significantly higher than those in the control group at the evaluated time points. Thus, the findings should be interpreted considering this characteristic of the experimental model.

Additional phenotypic markers, oxidative stress analyses, and intracellular signaling pathway assessments were not included and should be considered in future studies to provide a more comprehensive understanding of the mechanisms involved. The MTT assay and total protein quantification were used as complementary parameters, and not as isolated markers of cell viability, intracellular metabolism, or secretory function. In addition, the dexamethasone concentrations and PBM dosimetry were selected for an in vitro experimental model and should not be directly extrapolated to clinical practice. Although the PBM dosimetry was defined based on previous studies in J774 macrophages, future dose-response analyses may further clarify the observed effects. Similarly, because a commercial dexamethasone formulation was used, future studies with specific excipient controls may contribute to an even more precise evaluation of the observed effects. Additional studies using primary cells, additional inflammatory and phenotypic markers, and preclinical models are needed to further investigate these findings.

## Conclusion

Photobiomodulation, dexamethasone, and their combination modulated cellular and inflammatory responses in LPS/IFN-γ-stimulated J774 macrophages. The combination of PBM with low-dose dexamethasone was associated with modulation of TNF-α and IL-6 levels and a more favorable cellular response compared with dexamethasone alone. These findings suggest that PBM may contribute to improving the cellular response to low-dose dexamethasone in this in vitro model. Further studies using additional phenotypic and mechanistic markers are needed to confirm these effects and clarify their potential translational relevance.

## Data Availability

The datasets generated and/or analyzed during the present study are available from the corresponding author upon reasonable request. All relevant data supporting the findings of this study are included in the article.

## Abbreviations

ANOVA: Analysis of variance
DEXA: Dexamethasone
DMEM: Dulbecco’s Modified Eagle’s Medium
ELISA: Enzyme–linked immunosorbent assay
FBS: Fetal bovine serum
IFN: γ–Interferon–gamma
IL: 6–Interleukin 6
iNOS: Inducible nitric oxide synthase
LPS: Lipopolysaccharide
MAPK: Mitogen–activated protein kinase
MTT: 3–[4,5–dimethylthiazol–2–yl]–2,5–diphenyltetrazolium bromide
NF: κB–Nuclear factor kappa–light–chain–enhancer of activated B cells
PBS: Phosphate–buffered saline
PBM: Photobiomodulation
SEM: Standard error of the mean
TNF: α–Tumor necrosis factor alpha

## References

[1] Italiani P, Boraschi D (2014) From monocytes to M1/M2 macrophages: phenotypical vs. functional differentiation. Front Immunol 5:514. 10.3389/fimmu.2014.00514 25368618 10.3389/fimmu.2014.00514 PMC4201108

[2] Martinez FO, Sica A, Mantovani A, Locati M (2008) Macrophage activation and polarization. Front Biosci 13:453–461. 10.2741/2692 17981560 10.2741/2692

[3] Martinez FO, Gordon S (2014) The M1 and M2 paradigm of macrophage activation: time for reassessment. F1000Prime Rep 6:13. 10.12688/f1000research.3-13.v2 24669294 10.12703/P6-13 PMC3944738

[4] Chazaud B (2014) Macrophages: supportive cells for tissue repair and regeneration. Immunobiology 219(3):172–178. 10.1016/j.imbio.2013.09.001 24080029 10.1016/j.imbio.2013.09.001

[5] Tidball JG (2017) Regulation of muscle growth and regeneration by the immune system. Nat Rev Immunol 17(3):165–178. 10.1038/nri.2016.150 28163303 10.1038/nri.2016.150 PMC5452982

[6] Hamblin MR (2017) Mechanisms and applications of the anti-inflammatory effects of photobiomodulation. AIMS Biophys 4(3):337–361. 10.3934/biophy.2017.3.337 28748217 10.3934/biophy.2017.3.337 PMC5523874

[7] de Freitas LF, Hamblin MR (2016) Proposed mechanisms of photobiomodulation or low-level light therapy. IEEE J Sel Top Quantum Electron 22(3):7000417. 10.1109/JSTQE.2016.2561201 28070154 10.1109/JSTQE.2016.2561201 PMC5215870

[8] Karu TI (2010) Multiple roles of cytochrome c oxidase in mammalian cells under action of red and IR-A radiation. IUBMB Life 62(8):607–610. 10.1002/iub.359 20681024 10.1002/iub.359

[9] Woo KH, Kim YS, Lee SB, Chae SW (2025) Reprogramming Macrophage Phenotypes With Photobiomodulation for Improved Inflammation Control in ENT Organ Tissues. Clin Exp Otorhinolaryngol 18(1):1–13. 10.21053/ceo.2024.00836 39700888 10.21053/ceo.2024.00286 PMC11917203

[10] Dos Santos Malavazzi TC, Fernandes KPS, Lopez TCC, Rodrigues MFSD, Horliana ACRT, Bussadori SK et al (2023) Effects of invasive and non-invasive systemic photobiomodulation using low-level laser in experimental models: a systematic review. Lasers Med Sci 38(1):137. 10.1007/s10103-022-03604-5 37318623 10.1007/s10103-023-03799-x

[11] Fernandes KPS, Souza NHC, Mesquita-Ferrari RA, Silva DFT, Rocha LA, Alves AN et al (2015) Photobiomodulation with 660-nm and 780-nm laser on activated J774 macrophage-like cells: effect on M1 inflammatory markers. J Photochem Photobiol B 153:344–351. 10.1016/j.jphotobiol.2015.10.025 26519828 10.1016/j.jphotobiol.2015.10.015 PMC4674369

[12] Souza NHC, Mesquita-Ferrari RA, Rodrigues MFSD, da Silva DFT, Ribeiro BG, Alves AN et al (2018) Photobiomodulation and different macrophage phenotypes during muscle tissue repair. J Cell Mol Med 22(10):4922–4934. 10.1111/jcmm.13801 30024093 10.1111/jcmm.13757 PMC6156453

[13] Barnes PJ (1998) Anti-inflammatory actions of glucocorticoids: molecular mechanisms. Clin Sci 94(6):557–572. 10.1042/cs0940557 10.1042/cs0940557 9854452

[14] Rhen T, Cidlowski JA (2005) Antiinflammatory action of glucocorticoids—new mechanisms for old drugs. N Engl J Med 353(16):1711–1723. 10.1056/NEJMra050541 16236742 10.1056/NEJMra050541

[15] Kraaij MD, van der Kooij SW, Reinders MEJ, Koekkoek K, Rabelink TJ, van Kooten C et al (2011) Dexamethasone increases ROS production and T-cell suppressive capacity by anti-inflammatory macrophages. Mol Immunol 49(3):549–557. 10.1016/j.molimm.2011.08.014 22047959 10.1016/j.molimm.2011.10.002

[16] Souza NHC, Ferrari RAM, Silva DFT, Nunes FD, Bussadori SK, Fernandes KPS (2014) Effect of low-level laser therapy on the modulation of mitochondrial activity of macrophages. Braz J Phys Ther 18(4):308–314. 10.1590/bjpt-rbf.2014.0044 25076002 10.1590/bjpt-rbf.2014.0046 PMC4183262

[17] Ribeiro BG, Alves AN, Dos Santos LAD, Cantero TM, Fernandes KPS, Dias DS et al (2016) Red and infrared low-level laser therapy modulate oxidative stress during muscle repair. PLoS ONE 11(4):e0153618. 10.1371/journal.pone.0153618 27082964 10.1371/journal.pone.0153618 PMC4833286

[18] Calixto Da Silva DA, Silva Almeida FDLS, Ota TMN, Guimaraes DM, Fernandes KPS (2020) Effects of dexamethasone and photobiomodulation on pain, swelling, and quality of life after buccal fat pad removal: a clinical trial. J Oral Maxillofac Surg 78(11):1942.e1–1942.e9. 10.1016/j.joms.2020.06.022 10.1016/j.joms.2020.07.006 32768403

[19] de Brito Sousa K, Rodrigues MFSD, de Souza Santos D, Mesquita-Ferrari RA, Nunes FD et al (2020) de Fátima Teixeira da Silva D,. Differential expression of inflammatory and anti-inflammatory mediators by M1 and M2 macrophages after photobiomodulation with red or infrared lasers. Lasers Med Sci 35(2):337–343. 10.1007/s10103-019-02852-4 10.1007/s10103-019-02817-1 31152259

[20] Gavish L, Perez LS, Reissman P, Gertz SD (2008) Irradiation with 780 nm diode laser attenuates inflammatory cytokines but upregulates nitric oxide in lipopolysaccharide-stimulated macrophages: implications for the prevention of aneurysm progression. Lasers Surg Med 40(5):371–378. 10.1002/lsm.20635 18563774 10.1002/lsm.20635

[21] Berridge MV, Tan AS (1993) Characterization of the cellular reduction of 3-(4,5-dimethylthiazol-2-yl)-2,5-diphenyltetrazolium bromide (MTT): subcellular localization, substrate dependence, and involvement of mitochondrial electron transport in MTT reduction. Arch Biochem Biophys 303(2):474–482. 10.1006/abbi.1993.1311 8390225 10.1006/abbi.1993.1311

[22] Berridge MV, Herst PM, Tan AS (2005) Tetrazolium dyes as tools in cell biology: new insights into their cellular reduction. Biotechnol Annu Rev 11:127–152. 10.1016/S1387-2656(05)11004-7 16216776 10.1016/S1387-2656(05)11004-7

[23] Ghasemi M, Turnbull T, Sebastian S, Kempson I (2021) The MTT assay: utility, limitations, pitfalls, and interpretation in bulk and single-cell analysis. Int J Mol Sci 22(23):12827. 10.3390/ijms222312827 34884632 10.3390/ijms222312827 PMC8657538

